# Tumor Treating Fields (TTFields) Therapy Concomitant with Taxanes for Cancer Treatment

**DOI:** 10.3390/cancers15030636

**Published:** 2023-01-19

**Authors:** Ignace Vergote, Teresa Macarulla, Fred R. Hirsch, Carsten Hagemann, David Scott Miller

**Affiliations:** 1 Division of Gynecologic Oncology, Department of Gynecology and Obstetrics, University Hospitals, KU Leuven, Herestraat 49, European Union, 3000 Leuven, Belgium; 2 Vall d’Hebrón University Hospital, Vall d’Hebrón Institute of Oncology (VHIO), Passeig Vall d’Hebron 119-129, 08035 Barcelona, Spain; 3 Hospital Quirónsalud Barcelona, IOB Quiron, Plaça d’Alfonso Comín, 5, 08023 Barcelona, Spain; 4 Center of Excellence for Thoracic Oncology, The Tisch Cancer Institute, Icahn School of Medicine at Mount Sinai, 1 Gustave L. Levy Place, Box 1128, New York, NY 10029-6574, USA; 5 Section Experimental Neurosurgery, Department of Neurosurgery, University of Würzburg, Josef-Schneider-Str. 11, D-97080 Würzburg, Germany; 6 Division of Gynecologic Oncology, The University of Texas Southwestern Medical Center, 5323 Harry Hines Blvd., Dallas, TX 75390, USA

**Keywords:** Tumor Treating Fields (TTFields), taxanes, non-small cell lung cancer (NSCLC), ovarian cancer, pancreatic cancer, mechanism of action

## Abstract

**Simple Summary:**

Tumor Treating Fields (TTFields) are electric fields that prevent cancer cell survival and tumor growth, without impacting healthy cells. TTFields therapy is delivered to the tumor using arrays that are placed on the patient’s skin, surrounding the tumor site, without the need for invasive procedures. Taxanes are chemotherapies used to successfully treat several aggressive cancers and are associated with side effects such as neutropenia (low neutrophils) and peripheral neuropathy. Although taxanes are considered to be the standard of care for many cancers, there is a need to identify other treatments that can be used in combination, to enhance their effectiveness, without increasing the side effects. The preclinical (laboratory) and clinical (human) data summarized here suggest that TTFields therapy together with taxanes may be beneficial in the treatment of several cancers.

**Abstract:**

Non-small cell lung cancer, ovarian cancer, and pancreatic cancer all present with high morbidity and mortality. Systemic chemotherapies have historically been the cornerstone of standard of care (SOC) regimens for many cancers, but are associated with systemic toxicity. Multimodal treatment combinations can help improve patient outcomes; however, implementation is limited by additive toxicities and potential drug–drug interactions. As such, there is a high unmet need to develop additional therapies to enhance the efficacy of SOC treatments without increasing toxicity. Tumor Treating Fields (TTFields) are electric fields that exert physical forces to disrupt cellular processes critical for cancer cell viability and tumor progression. The therapy is locoregional and is delivered noninvasively to the tumor site via a portable medical device that consists of field generator and arrays that are placed on the patient’s skin. As a noninvasive treatment modality, TTFields therapy-related adverse events mainly consist of localized skin reactions, which are manageable with effective acute and prophylactic treatments. TTFields selectively target cancer cells through a multi-mechanistic approach without affecting healthy cells and tissues. Therefore, the application of TTFields therapy concomitant with other cancer treatments may lead to enhanced efficacy, with low risk of further systemic toxicity. In this review, we explore TTFields therapy concomitant with taxanes in both preclinical and clinical settings. The summarized data suggest that TTFields therapy concomitant with taxanes may be beneficial in the treatment of certain cancers.

## 1. Introduction

Cancer is a leading cause of death, accounting for one in six deaths worldwide [1]. In 2020, lung cancer was the most common cancer-related death (1.8 million deaths), followed by colorectal cancer and liver cancer [1]. Other aggressive solid tumors which pose treatment challenges and have high mortality rates include ovarian cancer [2] and pancreatic cancer [3].

Lung cancer has a 5-year survival rate ranging from 10% to 20% [4]. Non-small cell lung cancer (NSCLC) constitutes > 85% of all lung cancers [5], and 60–70% of patients present with advanced NSCLC at the time of diagnosis [4,6]. The multifaceted standard of care (SOC) for advanced NSCLC consists of molecular-targeted therapy or immunotherapy with or without chemotherapy or chemoradiation [7,8]. Neoadjuvant chemotherapy or concurrent chemoradiation consists of a platinum-based doublet (e.g., cisplatin and etoposide or carboplatin and paclitaxel) [7,8]. In the USA, neoadjuvant chemotherapy and immunotherapy (nivolumab) has recently been approved by the FDA [9,10].

Ovarian cancer has the highest mortality of all gynecological cancers [2]. Approximately 75% of patients present with stage III or IV cancer at the time of diagnosis according to the International Federation of Gynecology and Obstetrics cancer staging system [11,12], with a 5-year survival rate ranging from 17% to 39% [13]. The SOC in advanced ovarian cancer is surgery, carboplatin and paclitaxel with or without bevacizumab, and/or poly-ADP ribose polymerase inhibitor (PARPi) maintenance therapy [14,15].

Worldwide, pancreatic cancer is the 8th and 9th leading cause of death from cancer in men and women, respectively [3]; due to asymptomatic early stages, most patients are diagnosed at stage III or IV with a 5-year survival rate of approximately 9%. Due to its advanced stage presentation, a dearth of efficacious treatments, and frequent resistance to chemotherapy, pancreatic cancer is notoriously difficult to treat [16,17,18,19,20]. The SOC for unresectable advanced pancreatic cancer is chemotherapy, either FOLFIRINOX or gemcitabine with or without nab-paclitaxel, depending on the overall clinical presentation.

Combination treatment strategies are key in treating aggressive solid tumors, with the aim of enhancing efficacy in an additive or synergistic manner, reducing drug resistance, limiting metastatic potential, and hindering tumor growth [21]. Systemic treatments are frequently associated with unfavorable adverse events (AEs), thus novel combination treatment regimens should focus on improving efficacy without adding toxicity [21]. Due to the high prevalence and extremely poor prognoses, there is an unmet need to find effective and tolerable treatment regimens for NSCLC, ovarian cancer, and pancreatic cancer.

## 2. Tumor Treating Fields Overview

Tumor Treating Fields (TTFields) are electric fields that exert physical forces to disrupt cellular processes critical for cancer cell viability and tumor progression [22,23,24]. The electric fields range in frequency from 100 kHz to 500 kHz, which is too high to stimulate tissue, and too low to have ionizing or significant heating effects [25,26]. TTFields are delivered at a specific frequency based on the cancer cell type being targeted, allowing different types of cancers to be treated optimally [27]. The optimal TTFields frequency for NSCLC cells and pancreatic cancer cells is 150 kHz, and 200 kHz for ovarian cancer cells [28,29,30]. As TTFields selectively target the distinct properties of cancer cells such as division rate and morphology, healthy cells are largely unaffected [31,32,33].

**Figure 1:**
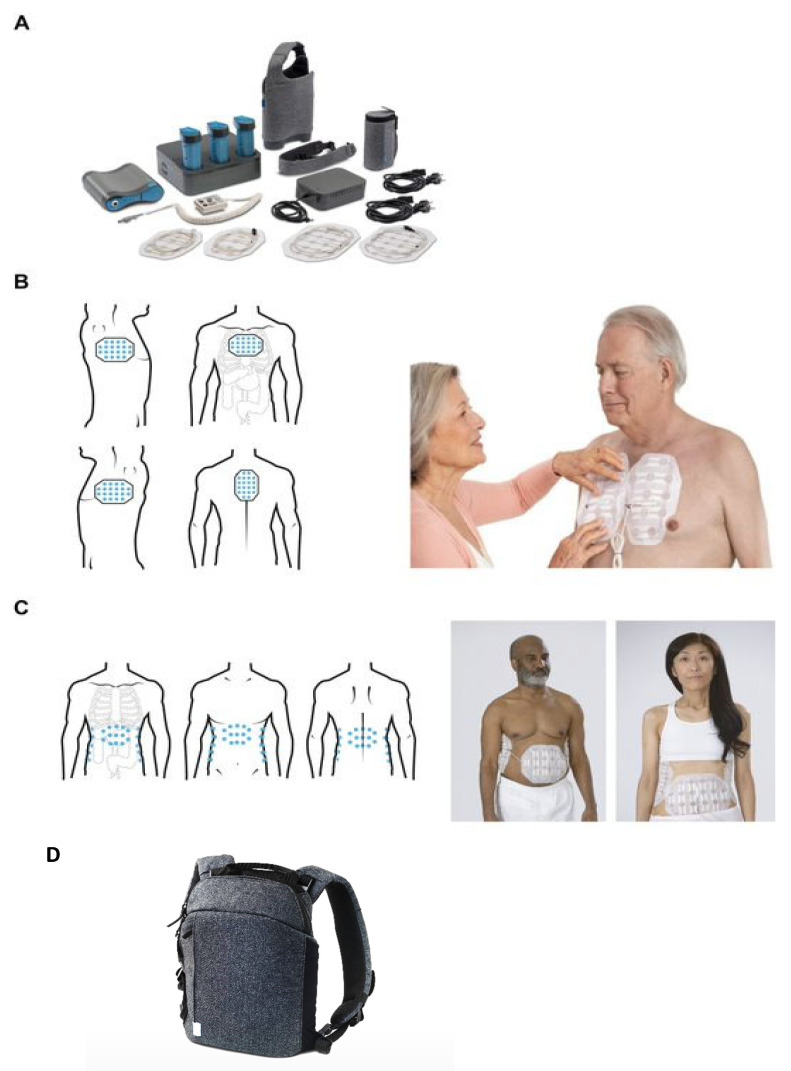
The Tumor Treating Fields (TTFields) medical device. (**A**) All components of the wearable medical device (NovoTTF-200T) that generates TTFields; example array layout * for (**B**) the torso and (**C**) the abdomen; (**D**) image of device within wearable backpack. Reused with permission from © 2023 Novocure GmbH-all rights reserved. The models depicted here are actors and not patients. * The exact location of the array placement is determined during treatment planning, based on tumor location.

TTFields therapy is delivered noninvasively to the tumor site via a portable medical device (NovoTTF-200; Figure 1) that consists of a field generator and arrays that are placed on the patient’s skin [34,35]. The magnitude of the anticancer effects of TTFields therapy is dependent on the frequency, intensity, and time of treatment [22,27,36], so it is recommended that TTFields therapy is used for at least 18 h/day to maximize treatment benefits [34,35,37]. The first-generation medical device (NovoTTF-100 device) weighed 6 lbs., however the second-generation medical device, NovoTTF-200, has been redesigned to weigh 2.7 lbs. to improve patient acceptance, satisfaction, and usage [38].

TTFields monotherapy is approved for recurrent glioblastoma (GBM) following results from the pivotal phase III EF-11 study (EF-11; NCT00379470) showing marked improvements in the safety profile and quality of life (QoL) compared to physician’s best choice [39]. TTFields therapy concomitant with temozolomide (TMZ) is approved for newly diagnosed GBM following positive results from the pivotal EF-14 study (EF-14; NCT00916409) whereby overall survival (OS) was extended by 4.9 months and progression free survival (PFS) by 2.7 months with TTFields therapy concomitant with TMZ vs. TMZ alone. Furthermore, there was no significant increase in systemic toxicity when TTFields therapy was added to TMZ [40,41].

TTFields therapy concomitant with pemetrexed and cisplatin/carboplatin is approved for unresectable malignant mesothelioma based on results from the phase II STELLAR study (EF-23; NCT02397928) wherein OS was improved compared to historical controls (18.2 vs. 12.1 months) with no increase in systemic toxicity [42,43]. Table 1 details the global approval status of TTFields therapy.

TTFields therapy has shown encouraging preliminary efficacy and a tolerable safety profile in several studies across a range of solid tumor types–including pancreatic, ovarian, and lung–when used concomitantly with systemic therapies [44,45,46,47]. Evaluation of TTFields therapy concomitant with a range of therapies in various solid tumors is ongoing.

## 3. The TTFields Mechanism of Action

**Figure 2:**
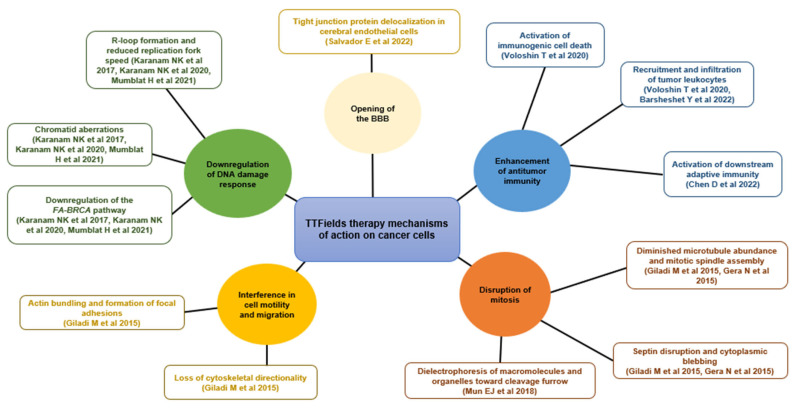
The Tumor Treating Fields multifaceted mechanism of action. BBB, blood–brain barrier; *FA-BRCA, Fanconi Anemia-BRCA* pathway [23,48,49,51,52,53,54,55,56,57,58].

TTFields target cancer cells via multiple mechanisms, physically disrupting processes important for cancer cells, which can lead to cell death (Figure 2) [22,24,25]. TTFields exert an anti-mitotic effect on cancer cells through induction of aberrant mitotic spindle formation during metaphase and disruption of the septin arrangement at the cleavage furrow, leading to cytoplasmic membrane blebbing, mitotic failure, and asymmetric chromosome segregation [48,49]. A recent study provided a theoretical mechanism of action that may explain this phenomenon: changes to the potential across tumor cell membranes could lead to an influx of Ca^2+^ ions and subsequent abnormal spindle formation and apoptosis [50].

TTFields have also been shown to downregulate genes important for DNA repair, such as the *Fanconi Anemia-BRCA* pathway [55,56,57]. Furthermore, TTFields-mediated increases in R-loop formation, decreases in replication fork speed, and increases in DNA double-strand breaks and chromatid aberrations, which can all result in apoptosis, have been observed [55].

TTFields enhance antitumor immune responses by inducing immunogenic cell death evidenced by release of high mobility group box 1 (HMGB1), initiation of the endoplasmic reticulum (ER) stress response, and translocation of calreticulin to the cell surface [52]. Moreover, TTFields upregulate autophagy [52,59,60,61,62] and promote an infiltration of activated tumor leukocytes in preclinical models [52]. Preclinical NSCLC data demonstrate that when TTFields treatment is used concomitantly with anti-programmed cell death protein-1 (anti-PD-1) and anti-cytotoxic T-lymphocyte-associated protein 4 (anti-CTLA-4) immunotherapies, there is an augmented antitumor effect with infiltration of tumor leukocytes and reduced tumor volumes [52,58]. Importantly, TTFields treatment does not reduce T-cell cytotoxicity [63]. TTFields also disrupt the nuclear envelope in vitro, activating stimulator of interferon genes (STING) and absent in melanoma 2 (AIM2) inflammasomes, which subsequently induces downstream adaptive immunity [53].

TTFields interfere with cancer cell motility and migration via disruption of the organization and dynamics of the microtubule network [24,60]. This leads to disruption of cellular polarity and formation of radial protrusions of peripheral actin filaments and focal adhesions, which can result in a loss of cytoskeletal directionality [24].

TTFields also transiently weaken the tight junctions between brain vascular endothelial cells that form the blood–brain barrier (BBB), through delocalization of tight junction proteins ZO-1 and claudin-5 [51]. As such, anticancer drugs can pass through the highly selective, semipermeable BBB more easily than typically expected, increasing local drug concentrations [51,64].

In vitro studies using GBM (U87-MG) cell lines showed that TTFields temporarily increase the susceptibility of cancer cells to therapeutics by altering the cell membrane structure and inducing pore formation to increase permeability. Importantly, this effect was reversed 24 h after the cessation of TTFields treatment, whilst healthy cells remained unaffected [65].

## 4. TTFields Therapy Concomitant with Systemic and Localized Anticancer Treatments

Due to the multimodal mechanism of action of TTFields therapy, efficacy of other systemic and localized anticancer treatments can be enhanced when utilized concomitantly with TTFields therapy, with a low risk of associated systemic toxicity [36,64]. As such TTFields therapy is in many cases an ideal candidate for treating aggressive solid tumors alongside SOC therapies.

### 4.1. TTFields Therapy Concomitant with Radiation

As with TTFields, radiation therapy causes DNA damage, resulting in cancer cell death [56], providing rationale for concomitant application. The addition of TTFields treatment to radiation therapy demonstrated enhanced efficacy in pancreatic [66], NSCLC [56], and GBM cell lines [67,68], as well as in murine colorectal models [69]. Moreover, the apoptotic effect was especially pronounced when TTFields treatment was applied prior to radiation therapy.

### 4.2. TTFields Therapy Concomitant with Immunotherapy

In line with the current evidence demonstrating the immunomodulatory effects of TTFields, concomitant use with immunotherapies invokes an additive effect both in vitro and in vivo, without diminishing T-cell-mediated cytotoxicity in vitro [52,63]. In vitro, TTFields treatment with anti-PD-1 demonstrated enhanced antitumor immunity in several cell lines [52]. In vivo, TTFields treatment with anti-PD-1 led to decreased lung tumor volume in mice, associated with increased ER stress and exposure of calreticulin [52]. Similarly, use of TTFields treatment with anti-PD-1 and anti-CTLA-4 also reduced tumor volumes in NSCLC mice models versus either agent alone, with an observed infiltration of tumor leukocytes [58]. Table 2 presents a summary of preclinical studies evaluating TTFields treatment with various immunotherapy agents.

### 4.3. TTFields Therapy Concomitant with Targeted Therapy

Concomitant use of TTFields treatment with PARPi produces synergistic anti-mitotic effects in human lung cancer cell lines (H1299 and H157), as both treatments induce cellular replication stress, cytotoxicity, and downstream apoptosis [55]. This effect is enhanced further when radiation is included. In vitro studies on GBM cell lines demonstrated that TTFields treatment concomitant with multi-kinase inhibitors led to inhibition of tumor cell motility, invasiveness, and angiogenesis, as well as an increase in autophagy [71]. Likewise, in hepatocellular carcinoma preclinical models TTFields treatment concomitant with sorafenib—a systemic small molecule multikinase inhibitor—led to a significant increase in cellular stress and subsequent apoptosis compared to either agent alone [62]. An overview of TTFields treatment with targeted therapies is shown in Table 3.

### 4.4. TTFields Therapy Concomitant with Chemotherapy

#### 4.4.1. TTFields Therapy Concomitant with Chemotherapy: Overview

Several clinical studies have evaluated the safety and efficacy of TTFields therapy used concomitantly with a range of chemotherapy agents, with findings demonstrating improvements in efficacy and a low risk of additive systemic toxicity (Table 3). TTFields therapy concomitant with the alkylating chemotherapeutic agent TMZ has demonstrated marked improvements in survival outcomes in patients with newly diagnosed GBM [41]. Although TMZ is able to pass through the BBB unaided, the aforementioned ability of TTFields to transiently weaken endothelial tight junctions may allow more TMZ to cross the BBB, increasing local drug concentrations and ultimately enhancing efficacy [51]. Additionally, TTFields induce reversible pore formation in GBM cells, which may facilitate localized increases of intracellular TMZ concentration, helping to improve treatment efficacy [53].

#### 4.4.2. TTFields Therapy Concomitant with Taxanes

Taxanes are microtubule-targeting antitumor agents that have been synthesized to effectively treat a wide range of aggressive solid tumors. Whilst taxanes have been a cornerstone cytotoxic treatment for the past 40 years, systemic AEs and drug resistance can present issues for patients [78,79]. Common dose-limiting AEs associated with taxanes include peripheral neuropathy, neutropenia, and fatigue [80,81,82,83].

The taxanes paclitaxel and docetaxel bind to β-tubulin subunits, leading to polymerization of highly stable microtubules and subsequent disruption to the microtubule organizing centre, cell cycle arrest, and eventual apoptosis [84]. Paclitaxel and docetaxel can also stimulate or inhibit downstream molecular pathways—preclinical studies with both treatments demonstrate nuclear translocation of transcription factors, increased caspase activation, and subsequent impaired cancer cell clonogenicity [79,85,86,87,88].

TTFields’ application leads to spindle disruption and apoptosis through decreased microtubule polymerization and subsequent increase in free tubulin [48,89]. On a molecular level, taxanes induce microtubule polymerization, facilitating growth of the polar protein chain and increasing its dipole moment—given that TTFields are electric fields that act on polar molecules, a longer microtubule may present an opportunity to exert more energy and force, thus enhancing mitotic catastrophe [89,90].

Therefore, as both TTFields therapy and taxanes target tubulin, causing mitotic catastrophe and cellular death, additive efficacy is observed when used together. Here, we report on preclinical and clinical studies investigating TTFields therapy concomitant with taxanes in various aggressive solid tumors.

## 5. TTFields Therapy Concomitant with Taxanes in NSCLC, Ovarian Cancer, and Pancreatic Cancer

### 5.1. TTFields Therapy Concomitant with Taxanes: NSCLC

Despite significant breakthroughs in the treatment of NSCLC, there are patients for whom immunotherapies are not suitable, or those who have progressed after immunotherapy treatment [91,92]. Effective and tolerable treatments that can be added to the existing SOC for advanced NSCLC, without additive systemic toxicity, are needed. Paclitaxel with or without carboplatin is a SOC in advanced NSCLC [7,93]; therefore, it is prudent to evaluate the feasibility of TTFields therapy with paclitaxel in NSCLC.

**Figure 3:**
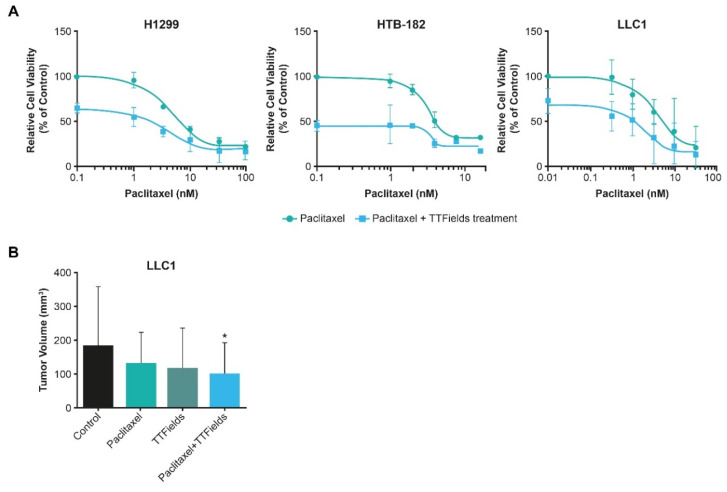
The effect of the TTFields treatment concomitant with paclitaxel on (**A**) cell viability in vitro on 2 human (H1299 and HTB-182) and 1 mouse (LLC1) cell line(s), and (**B**) tumor volume in vivo on the LLC1 mouse model. * *p* < 0.05 vs. control group. H1299 and HTB-182: human cell lines; LLC1: murine lung cancer cell line; TTFields: Tumor Treating Fields.

In vitro studies demonstrated a substantial reduction in viability of human (H1299 and HTB-182) and murine (LLC-1) NSCLC cells when TTFields (150 kHz) treatment was administered with paclitaxel vs. paclitaxel alone (Figure 3A) [30]. In vivo studies also illustrated that TTFields treatment concomitant with paclitaxel treatment reduced tumor size in murine NSCLC models, compared to tumors treated with paclitaxel alone (Figure 3B) [30]. These preclinical data highlight the efficacy benefit of TTFields treatment with paclitaxel, warranting further examination in clinical studies.

LUNAR is an ongoing pivotal, phase III randomized, open-label study (EF-24; NCT02973789) of TTFields therapy concurrent with SOC therapies (ICIs or docetaxel) for the treatment of stage IV NSCLC following platinum failure [94,95]. A total of 276 patients have been randomized 1:1 into the experimental arm (TTFields therapy and ICIs/docetaxel) or to the comparator of best SOC alone (ICIs/docetaxel) [94,95]. The primary endpoint is the OS of patients treated with TTFields therapy plus ICIs or docetaxel, vs. ICIs or docetaxel alone; key secondary endpoints include the OS of TTFields Therapy and docetaxel vs docetaxel alone, the OS of TTFields Therapy and ICI vs ICI alone, radiological response, PFS, QoL, TTFields therapy usage and the associated OS and PFS, and safety.

### 5.2. TTFields Therapy Concomitant with Taxanes: Ovarian Cancer

**Figure 4:**
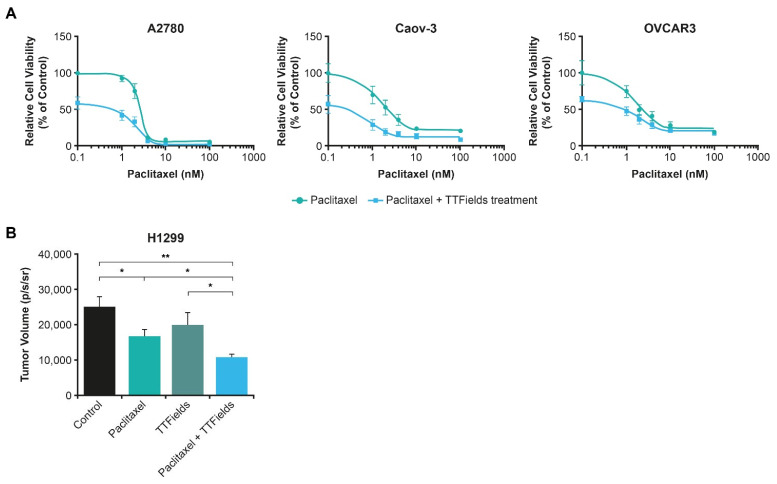
The efficacy of the TTFields treatment and paclitaxel on (**A**) human ovarian cell line (A2780, OVCAR3, Caov-3) count and (**B**) tumor volume for in vivo murine model figure. * *p* < 0.05 and ** *p* < 0.001. A2780: OVCAR3, Caov-3, human ovarian cancer cell lines; p/s/sr: photons per second per steradian; TTFields: Tumor Treating Fields.

Systemic SOC in advanced ovarian cancer consists of surgery, a platinum-based doublet (carboplatin and paclitaxel) with or without bevacizumab and eventual maintenance PARPi [15]. Preclinical data show a substantial reduction in ovarian cancer cell populations (A2780, OVCAR-3, Caov-3), as well as reduced cancer cell viability with the TTFields (200 kHz) treatment concomitant with paclitaxel, vs. paclitaxel alone (Figure 4A) [28]. Furthermore, in vivo murine models resulted in significantly lower tumor volume with the TTFields treatment concomitant with paclitaxel compared to sham-treated controls (*p* < 0.001) and mice treated with either paclitaxel alone (*p* < 0.05) or the TTFields treatment alone (*p* < 0.05) (Figure 4B) [28]. As a result of these promising preclinical data, paclitaxel and TTFields therapy has been evaluated in patients with ovarian cancer.

INNOVATE was a phase II, single-arm study assessing TTFields (200 kHz) therapy with paclitaxel in platinum-resistant ovarian cancer (PROC) (EF-22; NCT02244502) [47]. Overall, 31 heavily pre-treated patients (median age 60, range 45–77 years; median prior chemotherapy lines 4, range 1–11; median prior platinum lines 2, range 0–9) received weekly paclitaxel (80 mg/m^2^) concomitant with TTFields therapy [47]. The primary endpoint was safety; secondary endpoints included the OS, PFS, and response rate [47]. 

Twenty-six patients (84%) experienced mild-to-moderate (grade 1–2) TTFields therapy-related dermatitis and 2 patients (6%) experienced grade 3 TTFields therapy-related dermatitis; 1 patient (3%) permanently discontinued TTFields therapy due to dermatitis [47]. In terms of events likely associated with paclitaxel, grade 1–2 neutropenia was observed in 3% of patients, grade 3–4 neutropenia in 10% of patients, and grade 1–2 neuropathy in 45% of patients [47,84]. Overall, 32% of patients experienced serious AEs, all of which could be attributed to the underlying malignancy, previous or concomitant systemic therapy, or general health condition [47].

**Figure 5:**
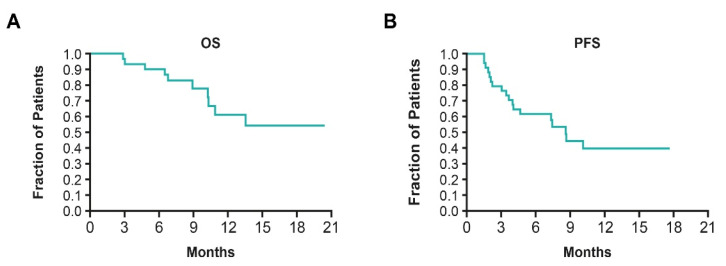
(**A**) OS and (**B**) PFS in heavily pre-treated patients with PROC receiving TTFields therapy concomitant with weekly paclitaxel [47]. OS: overall survival; PFS: progression-free survival; PROC: platinum-resistant ovarian cancer; TTFields: Tumor Treating Fields.

The median OS was not reached in INNOVATE (Figure 5A); OS rates at 6 and 12 months were 90% (95% confidence interval [CI], 72–97) and 61% (95% CI, 37–78), respectively. The median PFS was 8.9 months (95% CI, 4.7–not available) and partial responses were observed in 25% of patients (Figure 5B) [47]. Survival outcomes from INNOVATE were markedly improved compared to those cited in the literature, with one study reporting the OS and PFS for heavily pretreated patients in receipt of SOC therapies after their fourth relapse, as 6.2 months (95% CI, 5.1–7.7) and 4.4 months (95% CI, 3.7–4.0), respectively [96]. In INNOVATE, 71% of patients experienced a clinical benefit (stable disease or partial response). TTFields therapy usage was high with 77% of patients using it for 18 h/day in the first 3 months, and there were no paclitaxel dose reductions, suggesting good tolerability of this concomitant treatment regimen. The lack of additive systemic toxicity, encouraging survival outcomes, and high TTFields therapy usage provided a rationale for a larger phase III study (ENGOT-ov50/GOG-3029/INNOVATE-3).

ENGOT-ov50/GOG-3029/INNOVATE-3 (EF-28; NCT03940196) is a phase III prospective randomized study designed to evaluate the efficacy and safety of TTFields (200 kHz) therapy with weekly paclitaxel in patients with PROC. A total of 540 patients have been randomized 1:1 to either the experimental (TTFields therapy and weekly paclitaxel) or comparator arm (weekly paclitaxel alone) [97,98]. The primary endpoint is the OS, and key secondary endpoints include PFS, objective response rate, and QoL [97,98].

### 5.3. TTFields Therapy Concomitant with Taxanes: Pancreatic Cancer

**Figure 6:**
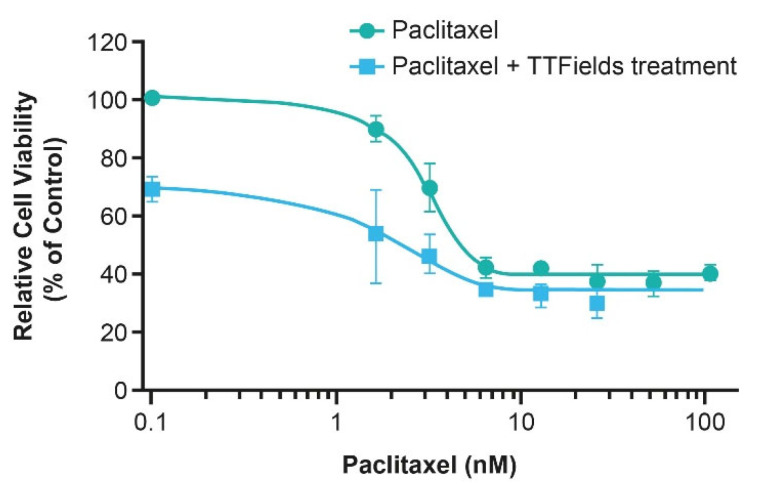
The effect of the TTFields treatment with paclitaxel on human pancreatic cancer cell line AsPC-1 cell viability. AsPC-1: human pancreatic cancer cell line; TTFields: Tumor Treating Fields.

Nab-paclitaxel (protein-bound paclitaxel) with gemcitabine is a SOC regimen in patients with pancreatic cancer [99], however poor survival necessitates an improvement in efficacy. Despite this need, it is challenging to identify treatments that can be administered concomitantly with nab-paclitaxel and gemcitabine, due to the risk of increasing potential systemic toxicities [100]. Given the low risk of systemic toxicity associated with TTFields therapy [23], evaluation of TTFields therapy concomitant with gemcitabine and nab-paclitaxel is warranted. In vitro application of TTFields (150 kHz) treatment with paclitaxel showed a substantial decrease in human pancreatic cancer cell (AsPC-1) count vs. paclitaxel alone (Figure 6) [29]. As such, clinical studies evaluating the use of TTFields therapy concomitant with paclitaxel in patients with locally advanced pancreatic cancer have been conducted.

PANOVA was a multicenter, open-label phase II study assessing TTFields (150 kHz) therapy concomitant with gemcitabine or TTFields therapy concomitant with gemcitabine and nab-paclitaxel in advanced pancreatic ductal adenocarcinoma (EF-20; NCT01971281) [44]. Overall, 40 patients were assigned 1:1 to receive TTFields therapy concomitant with weekly gemcitabine (1000 mg/m^2^) or TTFields therapy concomitant with weekly gemcitabine (1000 mg/m^2^) and nab-paclitaxel (125 mg/m^2^) [44]. The median age was 73 years (range 49–81) in the TTFields therapy and gemcitabine treatment arm, and 69 years (range 58–81) in the TTFields therapy, gemcitabine, and nab-paclitaxel treatment arm [44]. The primary endpoint was safety, and the secondary endpoints included TTFields therapy usage time, PFS, and OS [44].

There were no serious TTFields therapy-related AEs in either treatment arm; 21 (53%) patients reported TTFields therapy-related skin irritation, of which 7 were grade 3 (18%), and all were resolved following temporary reduction of daily TTFields therapy usage [44].

Furthermore, there was no increase in serious AEs overall compared to that expected with chemotherapy alone [44]. Peripheral neuropathy led to a treatment interruption of nab-paclitaxel in 20% of patients; however, all cases were grade < 4. Grade 3–4 neutropenia was reported in 35% of patients in the TTFields therapy, gemcitabine, and nab-paclitaxel treatment arm, and 20% of patients in the TTFields therapy and gemcitabine treatment arm [44]. The number of patients experiencing neuropathy and neutropenia is in line with previous literature on taxanes [82]. TTFields therapy usage time was 68–78% of the recommended average daily use of 18 h/day in both arms [44].

**Figure 7:**
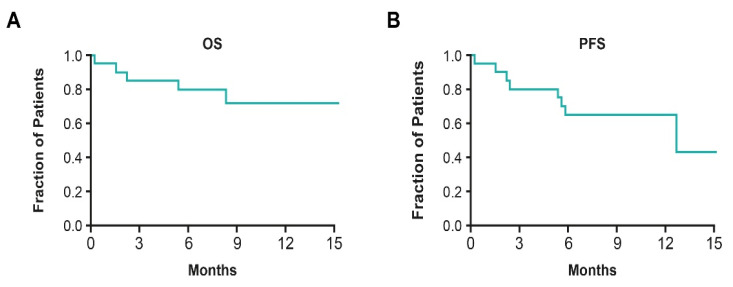
(**A**) OS and (**B**) PFS in patients with PDAC receiving TTFields therapy concomitant with gemcitabine and nab-paclitaxel [44]. OS: overall survival; PDAC: pancreatic duct adenocarcinoma; PFS: progression-free survival; TTFields: Tumor Treating Fields.

In the TTFields therapy, gemcitabine, and nab-paclitaxel arm, the median OS was not reached (Figure 7A); the 12-month survival rate was 72% (95% CI, 44–88) and median PFS was 12.7 months (95% CI, 5.4–NA) (Figure 7B) [44]. These results are improved compared to results from a similar phase II trial (NCT02301143), where the median OS and PFS for patients with pancreatic neoplasms treated with gemcitabine and nab-paclitaxel was 18.8 (90% CI, 15.0–24.0) and 10.9 months (90% CI, 9.3–11.6), respectively [101]. Based on these data, further evaluation of TTFields therapy concomitant with gemcitabine and nab-paclitaxel was warranted.

PANOVA-3 is a prospective, randomized, open-label, phase III study designed to assess the efficacy and safety of TTFields (150 kHz) therapy concomitant with gemcitabine and nab-paclitaxel compared to gemcitabine and nab-paclitaxel, in patients with treatment-naïve locally advanced pancreatic adenocarcinoma (EF-27; NCT03377491) [102,103]. The study aims to enroll 556 patients who will be randomized 1:1 to receive either TTFields therapy concomitant with gemcitabine and nab-paclitaxel or SOC (gemcitabine with nab-paclitaxel) [102,103]. The primary endpoint is OS; whereas, safety, PFS, objective response rate, and QoL are the key secondary endpoints [102,103].

## 6. TTFields Therapy Concomitant with Taxanes: Other Cancers

**Figure 8:**
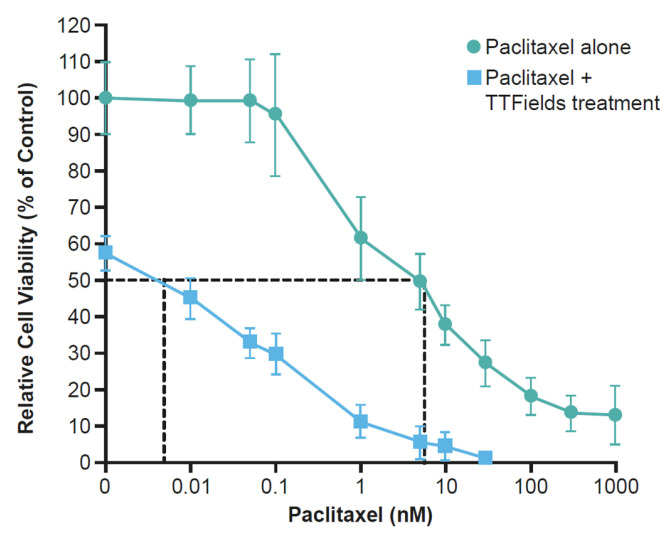
The effects of 72-h exposure of human breast carcinoma (MDA-MB-231) cells to paclitaxel alone at different concentrations and concomitant with TTFields treatment [90]. TTFields, Tumor Treating Fields.

Promising outcomes from clinical studies in ovarian and pancreatic cancer and preclinical studies in NSCLC highlight the possible benefit of TTFields therapy concomitant with taxanes in other aggressive solid tumors. Early preclinical studies of TTFields treatment concomitant with paclitaxel in human breast carcinoma (150 kHz) and human GBM (200 kHz) illustrated an additive effect with a synergistic tendency to reduce cell count (Figure 8) [27,90]. Furthermore, in cells isolated from NSCLC brain metastases, TTFields (150 kHz) treatment and paclitaxel resulted in a significantly diminished clonogenic potential vs. untreated controls or either treatment applied singularly [104]. Preclinical findings in NSCLC brain metastases and on the BBB provide rationale for investigation in a clinical setting. The phase III METIS study (EF-25; NCT02831959) investigating TTFields (150 kHz) therapy with SOC in patients with brain metastases from NSCLC is ongoing.

## 7. TTFields Therapy Concomitant with Taxanes: Summary of Clinical Efficacy and Safety

Taken together, the data demonstrate that, when utilized concomitantly with taxanes, TTFields act by preventing tumor cell proliferation, as well as sensitizing cells to the effects of taxanes [28,29,30,90]. In NSCLC, ovarian, and pancreatic cell lines, a reduction in cancer cell viability was observed when TTFields were applied with paclitaxel (Figure 3, Figure 4 and Figure 6) [28,29,30]. TTFields treatment together with paclitaxel led to a reduction in murine NSCLC and ovarian tumor volume vs. paclitaxel alone [28,30]. As taxanes are fundamental treatments for many solid tumors, the preclinical data highlight the need to further investigate the concomitant use of TTFields therapy with taxanes in a clinical setting.

Clinical data thus far have indicated that TTFields therapy does not increase systemic toxicity when used concomitantly with other chemotherapeutics or treatments in ovarian and pancreatic cancers [44,47]. The most common TTFields therapy-related AE was local skin irritation beneath arrays, consistent between different studies and different cancer types [44,47]. There were no serious TTFields therapy-related AEs in either study [44,47]. Although patients did experience neuropathy, commonly associated with taxanes, the addition of TTFields therapy did not exaggerate this effect [44,47]. Grade 3–4 neutropenia occurrence was low (10%) in the ovarian cancer study, but higher (35%) in the pancreatic cancer study, which may be explained by the addition of gemcitabine to the regimen, which is also associated with neutropenia [44,47].

## 8. Conclusions

Since their introduction, taxanes have been key treatments for a range of solid tumors. NSCLC, ovarian cancer, and pancreatic cancer have a high prevalence and are leading causes of cancer-related deaths. The SOC for these cancers is often associated with a poor AE profile and combinatorial treatment options should enhance the efficacy of established treatment options, without substantial additive systemic toxicity.

TTFields therapy is a non-invasive locoregional treatment, and an ideal candidate to be used with existing cancer therapies due to its multimodal mechanism of action and low risk of systemic toxicity. Preclinical data provided the rationale to investigate TTFields therapy concomitant with taxanes in clinical studies, which showed efficacy and tolerability in ovarian and pancreatic cancer, and further studies are ongoing. TTFields therapy concomitant with taxanes offers a promise of an innovative treatment regimen in these aggressive solid tumors.

## Figures and Tables

**Table 1:** A summary of the global approval status of TTFields therapy.

Disease	Country/Countries Where TTFields Therapy Is Approved
GBM	USA
	Canada
	China
	Hong Kong
	Japan
	Europe *^,†^
	Israel
	Australia
Pleural mesothelioma	USA
	Hong Kong
	Europe *

Approval status as of January 2023. * Approval in several European Union countries and Switzerland, which is not a European Union member. ^†^ Approved for grade 4 Glioma. GBM: glioblastoma; TTFields: Tumor Treating Fields.

**Table 2:** A summary of preclinical studies investigating TTFields treatment concomitant with immunotherapy or targeted therapy.

Study	Disease	Regimen	Key Findings
**Immunotherapies**			
Kim et al., 2021 [70]	Breast cancer	TTFields therapy concomitant with TRZ	TTFields treatment concomitant with TRZ enhanced penetration of TRZ after inducing apoptosis; TTFields overcame TRZ resistance in vivo and in vitro
Voloshin et al., 2020 [52]	NSCLC, colorectal cancer	TTFields therapy concomitant with anti–PD-1	Immunostimulatory effects from TTFields-induced cell death were observed; TTFields treatment utilized concomitantly with anti–PD-1 enhanced antitumor immunity and decreased tumor volume
Barsheshet et al., 2022 [58]	NSCLC	TTFields therapy concomitant with anti–PD-1 and anti-CTLA-4	TTFields treatment enhanced the immunostimulatory effect of anti–PD-1/anti-CTLA-4, causing tumor leukocyte infiltration and reduced tumor volume
**Targeted therapies**			
Davidi et al., 2022 [62]	HCC	TTFields therapy concomitant with sorafenib	Concomitant use of TTFields treatment and sorafenib led to augmented efficacy through increased cellular stress and apoptosis versus either agent alone
Jo et al., 2018 [71]	GBM	TTFields therapy concomitant with sorafenib	Sorafenib and TTFields treatment accelerated apoptosis via ROS generation; TTFields treatment and sorafenib significantly inhibited tumor cell motility, cell invasiveness, and angiogenesis
Kim et al., 2020 [72]	GBM	TTFields therapy concomitant with sorafenib	Sorafenib plus TTFields treatment significantly inhibited xenograft tumor growth; *STAT3* expression, linked to tumor progression, was also reduced

HCC: hepatocellular carcinoma; GBM: glioblastoma; PD-1: programmed death protein-1; NSCLC: non-small cell lung cancer; ROS: reactive oxygen species; *STAT3*: signal transducer and activator of transcription 3; TRZ: trastuzumab; TTFields: Tumor Treating Fields.

**Table 3:** A summary of clinical studies investigating TTFields therapy concomitant with chemotherapy.

Study	Disease	Phase	Regimen	Patients	Key Findings
**Pivotal studies**					
Stupp et al., 2017 EF-11; NCT00916409 [41]	ndGBM	III	TTFields therapy concomitant with TMZ	*N* = 695	**Median PFS, (95% CI) months** TTFields therapy with TMZ: 6.7 (6.1–8.1) TMZ: 4.0 (3.8–4.4) *p* < 0.001
Ceresoli et al., 2019 STELLAR; NCT02397928 [73]	Pleural mesothelioma	II	TTFields therapy concomitant with pemetrexed and cisplatin/carboplatin	*N* = 80	**Median OS, (95% CI) months** 18.2 months (12.1–25.8) *p* value NA
Rivera et al., 2019 PANOVA; NCT01971281 [44]	PDAC	II	TTFields therapy concomitant with gemcitabine TTFields therapy concomitant with gemcitabine and nab-paclitaxel	*N* = 40	**Safety** In each cohort, 85% reported grade ≥ 3 AEs No increase in SAEs vs. systemic chemotherapy alone
Vergote et al., 2018 INNOVATE; NCT02244502 [47]	PROC	II	TTFields therapy concomitant with paclitaxel	*N* = 31	**Safety** Overall, 55% reported grade ≥ 3% AEs No increase in SAEs vs. systemic chemotherapy alone
**Other studies**					
Lu et al., 2019 [74]	rGBM	RW	TTFields therapy concomitant with bevacizumab and irinotecan and TMZ TTFields therapy concomitant with bevacizumab-based chemotherapies *	*N* = 48	**Median OS, (95% CI) months** TTFields therapy concomitant with bevacizumab and irinotecan and TMZ: 32.5 (17.0–49.0) TTFields therapy concomitant with bevacizumab-based chemotherapies: * 17.8 (13.3–19.9) *p* < 0.05
Miller et al., 2022 NCT03477110 [75]	ndGBM	I	TTFields therapy concomitant with TMZ	*N* = 30	**Safety** No grade ≥ 3 AEs TTFields therapy-related AE reported Grade 1 and 2 skin toxicity reported in 73.3% and 10%, respectively
Garcia et al., 2018 [76]	rGBM	RW	TTFields therapy concomitant with TMZ	*N* = 21	**Safety** TMZ and TTFields therapy were well-tolerated, few AEs reported
Lazaridis et al., 2019 [77]	ndGBM	RW	TTFields therapy concomitant with lomustine and TMZ	*N* = 16	**Safety** Grade ≥ 3 hematologic and grade ≥ 3 hepatotoxic AEs were observed in 44% and 25% of patients, respectively

* Bevacizumab based chemotherapies either consist of bevacizumab + irinotecan or bevacizumab + procarbazine + lomustine. AE: adverse event; CI: confidence interval; ndGBM: newly diagnosed glioblastoma; OS: overall survival; PDAC: pancreatic ductal adenocarcinoma; PFS: progression free survival; PROC: platinum-resistant ovarian cancer; rGBM: recurrent glioblastoma; RW: real-world; SAE: serious adverse event; TTFields: Tumor Treating Fields; TMZ: temozolomide.
